# Nutrition information-seeking behavior among patients recovering at home after esophageal cancer surgery: a qualitative study

**DOI:** 10.3389/fnut.2026.1861145

**Published:** 2026-06-24

**Authors:** Xinhui Song, Lei Zhong, Huiyan Liao, Muxi Cheng, Qiuling Qin, Tong Wu, Mei Li

**Affiliations:** 1 Department of Nursing, Nanfang Hospital, Southern Medical University, Guangzhou, China; 2 School of Nursing, Southern Medical University, Guangzhou, Guangdong, China

**Keywords:** esophageal cancer, information-seeking behavior, nutrition, nutritional management, qualitative research

## Abstract

**Objective:**

Patients after esophageal cancer surgery often have unmet nutritional information needs during home rehabilitation. However, research on how these patients acquire, understand, and apply nutritional information in real-life contexts remains limited. This study aims to explore the nutritional information-seeking behaviors of post-esophagectomy patients during home rehabilitation, in order to provide evidence for the development of continuous and individualized nutritional information support strategies.

**Methods:**

A descriptive qualitative design with purposive sampling was employed in this study. Semi-structured interviews were conducted with 15 patients at a tertiary hospital in Guangdong Province, China, between November 2025 and January 2026. Thematic analysis was performed using NVivo 14.0 software to organize and interpret the data.

**Results:**

Four main themes emerged from the interviews: (1) multidimensional nutritional information needs; (2) Diversity of Information Sources; (3) barriers in the process of nutritional information seeking; and (4) behavioral patterns of nutritional information application.

**Conclusion:**

This study demonstrates that nutritional information seeking among patients recovering at home after esophageal cancer surgery is characterized by symptom-driven and fragmented patterns, with patients experiencing substantial difficulties in accessing, interpreting, and applying nutritional information. These findings highlight the need for continuous, individualized, and family-inclusive nutritional information support to strengthen long-term nutritional self-management during postoperative recovery.

## Introduction

Esophageal cancer (EC) is a common malignancy of the upper gastrointestinal tract. According to the 2022 global cancer statistics, its incidence ranks 11th and its mortality ranks 7th worldwide, and surgical resection remains the primary treatment modality (1, 2). With the promotion and application of enhanced recovery after surgery (ERAS), the length of hospital stay has been significantly reduced, and the postoperative rehabilitation process has gradually shifted to the out-of-hospital setting (3). However, most patients have not yet recovered normal digestive function and eating ability at the time of discharge, making the home phase a critical period for postoperative recovery and nutritional management. Due to the reconstruction of esophageal anatomy and changes in gastrointestinal function, patients after esophageal cancer surgery often experience restricted intake, impaired digestion and absorption, and related symptoms. The incidence of malnutrition can reach as high as 60–85% (4), which adversely affects the recovery process and quality of life. Therefore, continuous and individualized nutritional management is essential for promoting postoperative recovery. Compared with the in-hospital phase, patients receive relatively limited ongoing professional support during home rehabilitation after discharge. Their dietary management largely relies on individual self-adjustment based on the recovery process, which further increases dependence on their ability to access, understand, and apply information. Previous studies have shown that patients after esophageal cancer surgery have multi-level nutritional information needs (5). However, these needs are often not adequately met during the home rehabilitation phase, and patients continue to face insufficient access to information and a lack of continuity in support in real-life contexts. Nutritional literacy refers to an individual’s ability to understand, access, and apply basic nutrition-related information. At the same time, patients generally have low levels of nutritional literacy (6), with significant individual differences. This makes it difficult for them to effectively obtain, understand, and use complex information, which may affect the implementation of their nutritional management behaviors. Information-seeking behavior refers to the process by which individuals acquire and use information to meet specific needs (7). In the context of home rehabilitation after esophageal cancer surgery, patients need to continuously obtain and apply nutrition-related information regarding dietary adjustment and symptom management. However, in clinical practice, patients often encounter multiple barriers in the processes of information acquisition and utilization. Evidence indicates that cancer survivors generally have low levels of nutritional literacy, with particular deficits in analytical skills such as estimating nutrient intake (6, 8). In addition, with the rapid development of multi-source information environments and digital technologies, channels for accessing information have become increasingly diverse. Issues such as variable information quality and inconsistencies in content have become more prominent (9), increasing uncertainty in patients’ selection and use of information. In this context, the acquisition and application of nutritional information have become increasingly complex behavioral processes, which may have important implications for patients’ nutritional management. However, how patients actually navigate these complex information environments to meet their nutritional needs remains poorly understood.

At present, research on nutritional management in post-esophagectomy patients, both domestically and internationally, has mainly focused on evaluating the clinical effectiveness of nutritional interventions (10, 11). Although some studies have explored patients’ dietary-related experiences (12), little is known about how these patients actively seek, evaluate, and apply nutritional information during home-based recovery—particularly under the complex and often fragmented information environment they face after discharge. Based on this, this study adopts a qualitative research approach to explore the nutritional information-seeking behaviors of post-esophagectomy patients during home rehabilitation and the associated barriers, in order to provide evidence for optimizing discharge nutrition education strategies and developing individualized, continuous information support systems.

## Methods

This qualitative study was conducted at a tertiary hospital in Guangdong Province, China. The researchers explained the details of the study to the participants and obtained written consent from all participants who were willing to take part in the research. This study received ethical approval from the hospital’s ethics committee (No. NFEC-2025-611).

### Study design

The rationale for conducting this study stems from the lead researcher’s (SXH) dual role as both a nurse and clinical researcher in the Department of Thoracic Surgery, which provided the opportunity for in-depth clinical observations and insights into the topic. Additionally, other members of the research team have been engaged in the study of cancer patients and their care needs within their respective areas of expertise. Accordingly, a descriptive qualitative design based on thematic analysis was adopted for this study. Descriptive qualitative research is intended to explore participants’ thoughts, perceptions, and experiences related to a particular phenomenon (13). Given this objective, we considered a descriptive qualitative approach appropriate to explore esophageal cancer patients’ information-seeking behaviors, including the nutritional information needs, the channels through which they obtain information, barriers encountered during the process, and patterns of information application, during their home recovery period.

### Sample and setting

This study was conducted from November 2025 to January 2026 at a Grade A tertiary hospital in Guangdong Province. The inclusion criteria for patients were as follows: Diagnosed with esophageal cancer based on clinical evaluation and pathological examination; Patients who have undergone esophagectomy and are currently in the home-based recovery period; Attending regular follow-up appointments at the thoracic surgery outpatient clinic; Possessing normal communication and expression abilities; Voluntarily participating in this study and signing an informed consent form. Exclusion criteria included: Presence of severe postoperative complications; Concurrent severe cardiovascular or cerebrovascular disease or other malignant tumors; Presence of cognitive impairment or mental illness; Receiving palliative care or being unaware of one’s diagnosis. To gather diverse nutritional information from patients, purposive sampling was employed, with sample selection based on differences in patient age, educational level, Postoperative time (≤12 months), and family economic status. Interviews continued until no new themes or information emerged, indicating data saturation (14).

### Data collection

The principal investigator collected data through face-to-face, semi-structured interviews, which were recorded in their entirety. The time and location of the interviews were arranged according to the participants’ convenience. The interview guide was developed based on a review of relevant literature and the research team’s clinical experience, and was further revised and refined following preliminary interviews with two postoperative esophageal cancer patients. The final interview guide is as follows: (1) After being discharged from the hospital, what questions or concerns did you have regarding nutrition? Could you give a few specific examples? (2) What are your main sources of nutritional information? (3) Did you encounter any difficulties or inconveniences while obtaining this information? (4) After obtaining nutritional information, how did you apply it in practice? Could you provide an example? Data from the two pilot interview participants were excluded from the analysis. Prior to each interview, the researcher explained the study content, objectives, and significance to all participants, while strictly adhering to the principles of confidentiality and informed consent. The research team ensured that all interviews were conducted in a quiet, comfortable, and distraction-free environment. With participants’ permission, the interviews were audio-recorded. Throughout the interview process, the researchers respected the participants and sought to establish a trusting relationship, while avoiding leading or suggestive questioning to ensure that participants could express their genuine experiences and feelings voluntarily. In addition, interview techniques such as probing, paraphrasing, and clarification were employed to verify important information and confirm participants’ intended meanings, thereby ensuring the authenticity and comprehensiveness of the interview data. No repeat interviews were conducted. Interviews concluded when participants had no further information to add. Each interview lasted approximately 20–35 min and was fully audio-recorded.

### Data analysis

All interview recordings were transcribed verbatim within 48 h of completion. For participants with speech impairments or those who spoke in dialects during interviews, manual transcription was employed. The transcribed texts were imported into NVivo 14.0 software for text analysis to identify key issues. Analysis of the data was performed using the six-phase thematic analysis procedure proposed by Braun and Clarke (15). The thematic analysis followed these steps: (i) Two authors (SXH and ZL) repeatedly read the transcribed texts to familiarize themselves with the data content; (ii) The two authors independently developed and generated initial codes; (iii) Relevant data segments were grouped under each code and further synthesized into underlying themes; (iv) Themes were reviewed through repeated discussions among the authors; (v) Themes were defined and named; and (vi) A final report was drafted. In cases of disagreement, the researchers returned to the original transcripts and discussed the interpretations with other authors until consensus was reached.

### Reflexivity and rigor

Thematic analysis emphasizes the active reflexive role of researchers in the processes of coding and theme development. Throughout the study, reflexive journals were maintained to minimize potential researcher bias. In addition, rigor was enhanced by addressing transferability, dependability, confirmability, and credibility. Maximum variation sampling was adopted, and detailed descriptions of participant characteristics and the research process were provided to enhance transferability. The study was reported in accordance with the Consolidated Criteria for Reporting Qualitative Research (COREQ) guidelines to ensure dependability and credibility. Member checking was conducted both after the interviews and following the generation of the study findings. Furthermore, direct participant quotations were incorporated into the research report to strengthen confirmability.

### Ethical considerations

Throughout the study, we took careful measures to ensure informed consent and to protect participants’ privacy and confidentiality. Patients were explicitly informed that participation in the study was voluntary, that they could withdraw at any time, and that their withdrawal would not affect their subsequent medical care. To prevent the disclosure of personal information, all transcribed data were anonymized. Each participant was assigned a unique identification code (e.g., Patient 1 was designated as P1, Patient 2 as P2, and so on). The informed consent forms and audio recordings were securely stored in a locked cabinet in the principal investigator’s office, accessible only to members of the research team.

## Results

### Demographics of participants

Table 1 present the characteristics of the study participants. A total of 15 patients were invited to participate in this study. The total interview duration of this study was 465 min. The patients ranged in age from 49 to 72 years, with a mean age of 62 years. The majority had a monthly income at a mid-to-low level.

**Table 1:** Demographic and clinical characteristics of patients.

Participant	Gender	Age (years)	Educational level	Residence	Monthly household income per capita (Chinese yuan)	Pathological stage	Postoperative time (months)
P1	Male	58	Junior High School	Rural	3,000–5,000	II	8
P2	Female	62	no formal education	Rural	3,000–5,000	IV	4
P3	Male	49	Junior High School	Urban	5,000-10,000	III	3
P4	Male	64	Senior high school	Urban	5,000-10,000	III	1
P5	Male	55	Primary school	Rural	3,000–5,000	II	2
P6	Male	56	Primary school	Rural	3,000–5,000	III	4
P7	Female	63	Primary school	Rural	3,000–5,000	I	3
P8	Male	70	Primary school	Rural	3,000–5,000	I	5
P9	Male	60	Primary school	Urban	3,000–5,000	III	3
P10	Male	67	Senior high school	Urban	5,000-10,000	I	3
P11	Male	58	Senior high school	Rural	3,000–5,000	I	6
P12	Male	71	Junior High School	Rural	3,000–5,000	II	2
P13	Female	72	no formal education	Rural	3,000–5,000	III	3
P14	Male	62	Junior High School	Rural	3,000–5,000	I	2
P15	Male	63	Primary school	Rural	3,000–5,000	III	3

### Theme 1: The multidimensional nature of information needs

#### Subtheme 1: Need for basic dietary knowledge

During home rehabilitation after surgery, patients undergo reconstruction of the digestive tract and need to adapt their diet gradually from liquid to soft foods and then to a regular diet. Most participants lacked basic scientific understanding of “what to eat” and “how to eat” during this transitional phase. They commonly expressed specific needs for knowledge regarding food properties and types.

*“I was told by the doctor to transition gradually. The discharge summary mentioned liquid and semi-liquid diets, but I did not really understand what that meant.”* (P4)

*“I don’t know which foods are considered high in protein. Does egg count? Also, I want to know whether we can take Ganoderma as a supplement.”* (P8)

#### Subtheme 2: Need for practical skills

Some patients reported that although they were aware of basic dietary principles, they still did not know how to implement them in practice. This was particularly evident among patients requiring home enteral nutrition, who expressed an urgent need for clear operational guidance on tube feeding procedures.

*“I don’t know how long each feeding of the nutritional solution should take. The nurse didn’t explain it clearly. Would it be better to buy a feeding pump?”* (P11)

*“Even after grinding the medication, there are still some particles. Will this clog the tube if I administer it like this? Can homemade soup be given through the tube?”* (P2)

#### Subtheme 3: Need for symptom management

Post-esophagectomy patients commonly experience a range of nutrition-related symptoms, including early satiety, postprandial bloating, reflux, diarrhea, swallowing discomfort, and loss of appetite. Almost all participants reported experiencing such symptoms and expressed a need for strategies to relieve them as well as explanations for their causes.

*“Sometimes I feel a blockage when eating, and occasionally I choke. I’m worried that it might be a stricture. I mentioned it to the doctor, and they said it’s normal and will gradually improve, but I’m still worried it could be a stricture.”* (P11)

*“Now I get diarrhea whenever I drink milk. It never happened before, but it started after the treatment. I don’t know why.”* (P8)

*“After chemotherapy, I lost my appetite. I don’t feel like eating anything. Is there any way to relieve this?”* (P15)

### Theme 2: Diversity of Information Sources

#### Subtheme 1: Healthcare-provider-based information seeking

Some patients regarded healthcare professionals as the most trustworthy source of information. When encountering nutrition-related problems, they tended to seek answers from doctors or nurses and relied on their advice as the primary basis for nutritional management.

*“I didn’t look up any additional information online. I only trust the advice given by my doctor; what the doctor tells me must be important.”* (P2)

*“Whenever I have any questions, I just ask my attending physician. I don’t go online to check, because you never know whether what’s on the internet is true or false.”* (P11)

#### Subtheme 2: Family-agent-based information seeking

During home-based recovery, some patients relied on family members to search for, filter, and interpret relevant information on their behalf. Particularly when experiencing physical discomfort or diet-related problems, family members often became their primary source of information acquisition and decision-making support.

*“Whenever I feel unwell at home, I tell my daughter. I don’t know how to look things up, so I ask her to help me find out the possible causes and how to deal with them.”* (P3)

*“I rely on my wife for everything about nutrition. I don’t understand anything about it. She’s in charge of cooking. Whatever she cooks, I eat, and she tells me what I can’t eat.”* (P9)

#### Subtheme 3: Peer-support–based information seeking

Some patients actively sought communication with peers or fellow patients to obtain practical dietary transition strategies, food selection advice, and emotional support.

*“A fellow patient said eating mudfish could improve protein intake, so I tried it and asked my family to buy some as well.”* (P3)

*“I didn’t know how to eat after surgery. A fellow patient shared her daily eating pattern and advised me to take it gradually.”* (P6)

#### Subtheme 4: Internet-based information seeking

Faced with delayed clinical communication after discharge, some patients relied on internet search engines or AI-based conversational tools to support immediate dietary decision-making. These digital tools were used to compensate for limited access to professional guidance.

*“After discharge, I usually search online first when I have questions. Technology is very advanced now, you can find almost anything online. If I still don’t understand, then I ask the doctor.”* (P9)

*“When I’m unsure whether I can eat something, I ask Doubao. Its answers seem quite well-supported.”* (P13)

*“When scrolling Douyin, I often see videos about postoperative diet and nutritional care. Some are difficult to understand, so I may check again or seek clarification.”* (P10).

### Theme 3: Barriers in the process of nutritional information seeking

#### Subtheme 1: Limited awareness of nutritional issues

Some patients failed to recognize postoperative symptoms as nutrition-related issues. Although they experienced reduced food intake, swallowing discomfort, or weight loss, they often attributed these conditions to normal postoperative recovery. In addition, some patients had a vague understanding of nutritional management, simply equating it with “eating more” or maintaining a “light diet,” and lacked a systematic understanding of nutrition-related problems.

*“I did not ask the doctor about acid reflux. They said it is normal because I no longer have the anatomical structure to prevent reflux.”* (P8)

*“I definitely eat less than before now. My stomach has become smaller, so I cannot eat too much.”* (P9)

*“As for eating, I now keep it light and have soups every day to supplement nutrition.”* (P11)

#### Subtheme 2: Limited access to professional nutritional information

During the process of seeking nutritional information, some patients reported difficulty in obtaining continuous and systematic professional guidance. After discharge, patients had limited access to healthcare professionals and could only receive fragmented advice during follow-up visits. At the same time, professional and authoritative nutritional information from other sources was relatively limited.

*“When I was in the hospital, someone taught me. After I went home, no one followed up, and I did not know who to ask when I had problems.”* (P4)

*“Sometimes I have questions at home and want to ask a doctor, but it’s inconvenient to go to the hospital. I’ not sure whether online information is reliable, so if it’s not serious, I just deal with it myself.”* (P11)

#### Subtheme 3: Inconsistency among multiple information sources

Although patients are exposed to various types of nutritional information, they lack the ability to discern it and find it difficult to make scientific judgments from conflicting data, thereby further exacerbating their sense of uncertainty in self-nutritional management.

*“The doctor* advised *me not to drink soup but to eat the meat, saying that the nutrition is in the meat, not the soup. But in our usual habits, we only drink the soup and throw away the meat.”* (P6)

*“During chemotherapy, I wondered whether I could take traditional Chinese medicine for conditioning. One doctor said no because it would burden the liver and kidneys, while others said their doctors recommended it for gastrointestinal regulation.”* (P2)

*“At discharge, the nurse gave me a discharge summary, which only said ‘small, frequent meals and high-protein diet.’ I did not know how many meals to eat per day or how much to eat. I asked my son to search online, but we could not find a precise answer. I could not reach any professional, so in the end I could only estimate it myself.”* (P14)

### Theme 4: Behavioral patterns of information application

#### Subtheme 1: Passive adherence

Some patients exhibited passive adherence during the process of information adoption. When patients had a high level of trust in the information source, especially guidance from healthcare professionals, they tended to follow the advice as instructed, with little questioning or inquiry into the underlying rationale.

*“The doctor said I should eat high-protein foods every day, such as eggs, so I eat eggs daily to supplement protein.”* (P5)

*“I saw that some patients take traditional Chinese medicine after surgery, but my doctor said not to, so I didn’t take it.”* (P8)

#### Subtheme 2: Individualized adjustment

Some patients did not fully follow the recommendations during information adoption but instead selected and adapted them according to their own circumstances. Based on personal preferences, lifestyle habits, or tolerance, they selectively adopted or modified the information they received.

*“The doctor said I should increase protein intake. I tried the nutritional powder a few times, but I didn’t like it, so I stopped. I think drinking milk every day is enough to supplement protein.”* (P3)

*“I saw online that some patients use a wedge pillow to prevent reflux, but I couldn’t get used to it, so I used two regular pillows instead. I feel it works the same.”* (P10)

#### Subtheme 3: Discontinuation of implementation

Some patients, despite receiving nutritional information or healthcare professionals’ recommendations, ultimately discontinued implementation due to difficulties in understanding the information, complexity of procedures, personal taste preferences, or physical discomfort experienced during attempts.

*“The information online is too complicated, like protein and carbohydrate ratios. I don’t understand it, so I just ignore it.”* (P4)

*“They suggested blending food into a paste, but I found it too troublesome and unappetizing, so I preferred to eat normally.”* (P12)

*“The doctor said I could try eating rice or steamed buns, but when I eat rice, it got stuck in my throat. I can’t swallow it, so I don’t dare eat rice anymore. I don’t like steamed buns either.”* (P9)

## Discussion

The study found that postoperative patients with esophageal cancer exhibited diverse nutritional information needs, including basic dietary knowledge, practical dietary management skills, and symptom-related coping strategies. This finding is consistent with previous studies reporting substantial unmet nutritional information needs among patients with esophageal cancer following surgery (5, 16). Prior research suggests that when individuals perceive information gaps or uncertainty, they tend to actively seek information to support health-related decision-making (17, 18). However, in the present study, patients’ nutritional information needs were largely symptom-oriented and fragmented, with relatively limited attention given to systematic nutritional management issues such as energy requirement assessment or nutrient composition. This may be related to the fact that patients primarily focused on managing postoperative eating-related symptoms, including dysphagia, reflux, and early satiety, while lacking a continuous and structured understanding of nutritional management. Notably, nutritional information seeking was often initiated only after the onset of symptoms or problems, reflecting a certain degree of passivity and delay. Although symptom-driven information seeking may help patients cope with immediate health concerns, it may also delay early identification of nutritional risks and timely intervention. Previous studies have reported that cancer survivors frequently rely on symptom-driven or reactive self-management approaches during long-term recovery. In these cases, supportive actions are often initiated only after symptoms become disruptive to daily life (9, 19). These findings suggest that nutritional support for postoperative patients with esophageal cancer should extend beyond one-time discharge education and place greater emphasis on continuous information support throughout home-based recovery, thereby facilitating the gradual development of systematic nutritional management awareness.

The study further found that patients obtained nutritional information from multiple sources, including healthcare professionals, family members, peers, and internet-based platforms, which is consistent with previous findings (12). Among these, healthcare professionals remained the most trusted source of information, and patients expressed strong expectations for more timely and continuous professional nutritional guidance. However, patients reported that the nutritional information provided by clinicians was often standardized and principle-based. This may be because physicians primarily focus on disease treatment and have limited time and specialized training in detailed nutritional management (19). Previous studies have also indicated that clinicians’ involvement in nutritional counseling is limited, and the information provided is often general in nature, making it difficult to meet individualized patient needs (20). These findings suggest that multidisciplinary collaboration should be strengthened in clinical practice, with greater involvement of dietitians and specialist nurses to provide more targeted and actionable nutritional guidance, thereby improving the quality of information provision and patient outcomes.

In addition to professional information sources, most participants relied heavily on family members to obtain and interpret nutritional information, reflecting a prominent pattern of proxy health information seeking. Although this phenomenon may partly relate to the demographic characteristics of the sample: primarily middle-aged and older adults from rural areas with relatively low educational attainment, it may also reflect the family-centered nature of healthcare decision-making within the Chinese sociocultural context (21, 22). For many participants, nutritional information seeking was not solely an individual activity but a family-mediated process in which caregivers participated in searching, filtering, interpreting, and retransmitting health information. Previous studies similarly suggest that family caregivers acting as proxy information seekers may help patients overcome digital and health literacy barriers, facilitate access to health information, and provide emotional and practical support throughout disease management (23). However, information may undergo selective filtering, misunderstanding, or reinterpretation during transmission between caregivers and patients, thereby influencing patients’ comprehension and application of nutritional recommendations. Furthermore, excessive reliance on family-mediated information support might unintentionally limit opportunities for patients to directly engage with professional information and gradually develop independent self-management capacity. These findings highlight the importance of extending nutritional information support beyond patients alone and incorporating family caregivers into discharge preparation and transitional care programs. In particular, nutritional education and information literacy interventions targeting caregivers may improve the consistency, accuracy, and effectiveness of information communication among rural middle-aged and older populations recovering from esophageal cancer surgery. Peer support also emerged as an important source of nutritional information. Experience sharing among patients appeared to alleviate uncertainty and provide practical coping strategies during postoperative recovery. However, such information was largely based on individual experiences, and its scientific validity and applicability varied considerably. When experiential information lacks professional verification, it may further increase uncertainty in health-related decision-making (24). Therefore, while acknowledging the supportive value of peer interaction, healthcare professionals should also help patients develop the ability to critically appraise experiential information and explore professionally supported peer-support models that integrate experiential sharing with evidence-based guidance. Notably, only a minority of participants actively sought nutritional information through internet-based platforms. Although most patients owned smartphones, they more commonly engaged in passive browsing rather than active information searching. This may be associated with older age and limited eHealth literacy among participants. The present findings further suggest that the effective use of digital health information depends not only on the availability of information channels but also on patients’ abilities to search for, evaluate, and apply information appropriately (24, 25). Therefore, alongside the promotion of digital health services, greater attention should be paid to improving patients’ eHealth literacy to facilitate effective utilization of nutritional information.

During the information-seeking process, patients commonly encountered multiple barriers, including insufficient recognition of nutritional problems, limited access to professional nutritional information, and inconsistency across multiple information sources. This study found that most postoperative esophageal cancer patients experienced symptoms such as dysphagia and early satiety after discharge, however, these symptoms were often perceived as routine post-discharge expectations rather than health problems requiring active reporting or intervention. This is consistent with previous findings that individuals are unlikely to engage in information seeking when they do not perceive a need for information (26, 27). A qualitative study by Santin et al. on long-term recovery after esophagectomy further reported that patients rarely mentioned symptoms spontaneously during follow-up unless explicitly prompted, and this reactive pattern of symptom reporting further limited opportunities for timely problem identification and intervention (28). Patients’s understanding of nutritional management was often restricted to experiential descriptions or vague concepts, lacking a systematic cognitive framework. As a result, they were unable to establish clear logical links between specific symptoms and corresponding nutritional interventions. These findings suggest that healthcare professionals should actively assess patients’ level of nutritional knowledge and self-management capacity during nutritional education, and guide them to develop a clearer cognitive association between disease manifestations and nutritional management. This may help reduce misattribution of symptoms and facilitate timely initiation of information-seeking behaviors.

This study further revealed that although most patients wished to obtain professional and reliable nutritional information, professional support was disrupted after discharge. Moreover, in the highly abundant online information environment, patients lacked systematic access pathways and the ability to judge source credibility, making it difficult for them to effectively identify professional nutritional information and thereby increasing uncertainty in the information-seeking process. The scoping review by Treadgold and colleagues further revealed the risks of this dilemma: evidence of low-quality information and misinformation in online peer support groups often outweighed that of high-quality information, and existing health information quality appraisal tools are not designed for peer-generated content (24). It is recommended that healthcare providers offer patients clearly identifiable authoritative information sources and simplified information navigation methods at discharge, thereby improving the efficiency of acquiring and using professional nutritional information by directly pointing to reliable resources and reducing the difficulty of information filtering.

In addition, patients encountered considerable difficulties in interpreting and applying nutritional information, primarily due to inconsistencies across multiple information sources. Divergent and sometimes conflicting recommendations regarding dietary content, feeding practices, and nutritional supplementation contributed to confusion and hesitation during information integration and decision-making, thereby hindering subsequent adoption and implementation. Most participants in this study were from Guangdong, a region with deeply rooted dietary traditions, including the consumption of slow-simmered nourishing soups and the practice of consuming soup in place of meat. In addition, the use of herbal remedies for postoperative convalescence was also common in this population. These culturally embedded practices often conflict with contemporary evidence-based nutritional guidelines, leading to cognitive dissonance when patients are exposed to multiple sources of information.

Existing literature has indicated that inconsistency between cultural beliefs and medical recommendations is a key determinant of reduced adherence and suboptimal health-related decision-making (29). When patients receive contradictory information from family members, social media, and peer networks compared with that provided by healthcare professionals, they frequently experience decisional uncertainty. Owing to limited capacity for critical appraisal and information validation, patients are often unable to reconcile these discrepancies independently. Therefore, during discharge education and transitional care, nurses should proactively identify non-professional information sources that patients may encounter and engage in open, non-judgmental communication to elicit patients’ pre-existing beliefs. In addition, clinicians should clarify the context-specific applicability of different nutritional recommendations across disease stages, symptom profiles, and individual variability, rather than providing uniform prescriptive advice. Healthcare providers should first acknowledge the legitimacy of culturally rooted dietary beliefs and then introduce evidence-based guidance through a gradual, negotiated approach to reduce resistance and improve acceptance.

Regarding information utilization, not all patients successfully translated acquired nutritional information into appropriate self-management behaviors. Three distinct patterns of information application were identified: passive adherence, individualized adaptation, and non-implementation. These patterns reflect varying degrees of patient engagement and discontinuities in the translation from information acquisition to behavioral execution. Patients demonstrating passive adherence tended not to question the underlying rationale of nutritional recommendations and followed instructions mechanically, particularly when advice was delivered by healthcare professionals. The advantage of this pattern lies in the high level of trust in clinicians, which facilitates efficient transmission and initial behavioral compliance. However, excessive reliance on passive adherence may also pose risks. Nutritional recommendations in clinical practice are often generalized, whereas postoperative recovery trajectories vary substantially across individuals. Moreover, patients with strong trust in clinicians may be less likely to report adverse experiences, out of concern for challenging authority or troubling their physicians, which may lead to inaccurate clinical assessment of intervention effectiveness. Limited understanding of the rationale underlying dietary recommendations may further constrain patients’ autonomy in decision-making and reduce self-advocacy in disease management (30).

In contrast, patients exhibiting individualized adaptation actively reprocessed nutritional information by integrating prior experiences, habitual dietary patterns, and subjective bodily perceptions. This behavior reflects a degree of patient agency in health-related decision-making. Information use represents a critical transitional stage linking information seeking to behavioral outcomes, involving evaluation, integration, and application (31). However, such experience-based adaptation is not always aligned with evidence-based nutritional principles. Prior studies have shown that patient-led modifications may diverge from professional recommendations and potentially compromise nutritional status and recovery outcomes (32, 33). Given the lack of objective monitoring and clinical evaluation, these adaptations are largely guided by subjective perceptions, increasing the risk of suboptimal nutritional management (32). This suggests that healthcare professionals should recognize and encourage patients’ motivation to adapt while also guiding them to evaluate the appropriateness of their strategies.

Furthermore, some patients abandoned nutritional recommendations due to difficulties in comprehension, operational complexity, or prior negative feeding experiences, suggesting a breakdown between information acquisition and implementation. This may be attributed to the combined physiological stress and psychological burden experienced by postoperative esophageal cancer patients. Highly technical or insufficiently operationalized information increases cognitive load and reduces adherence intention. Negative feeding experiences further reinforce avoidance behaviors. Consistent with prior research, when health information exceeds an individual’s processing capacity, information avoidance or behavioral disengagement is a common coping response (34, 35). Wilson further emphasizes that information use represents the final and most critical stage of the information behavior process, determining whether information is ultimately translated into health behavior (36). Therefore, clinical practice should not only focus on information delivery but also emphasize the usability and implementability of nutritional guidance. Nurses should continuously assess patients’ real-world experiences following information provision, identify barriers related to comprehension or operational difficulty, and reduce cognitive burden through optimized information design, such as multimodal delivery and stepwise instruction. In addition, within the framework of shared decision-making and respecting patient autonomy, alternative nutritional strategies and ongoing follow-up support should be provided to bridge the gap between information acquisition and behavioral execution.

### Strengths and limitations of study

This study focuses on the nutritional information-seeking behaviors of patients with esophageal cancer during the postoperative home recovery phase. By exploring patients’ nutritional information needs, information acquisition pathways, and information application behaviors from the patient perspective, the findings may provide a basis for the development of targeted nutritional information support interventions in the future. However, this study has certain limitations. First, the participants were recruited from a single tertiary hospital in Guangdong Province, China. Differences in healthcare resource allocation, accessibility of nutritional support, and regional dietary culture across different areas may limit the transferability of the findings. In addition, the sample mainly consisted of middle-aged and older male patients from rural areas with relatively low educational and middle-to-low income levels, which may not adequately capture the information-seeking patterns of urban residents, highly educated individuals, younger patients, or female patients. Second, the interview data were primarily based on patient self-report and may therefore be subject to recall bias. Moreover, the cross-sectional nature of the interviews limited the ability to comprehensively capture the dynamic changes in patients’ nutritional information-seeking behaviors over time, fully capture dynamic changes in self-management barriers over time. This study did not incorporate pathological stage or postoperative time as contextual variables in the analysis, which may have masked variations in information needs. Finally, patients were the sole source of information in this study. Perspectives from family caregivers were not included, nor were the findings cross-validated with clinical records. Family caregivers often play an important intermediary role in patients’ nutritional information acquisition; however, this study did not directly explore caregivers’ information behaviors or their interaction patterns with patients, making it difficult to comprehensively reflect the alignment of needs among multiple stakeholders involved in the nutritional information-seeking process.

### Future research direction

Based on the findings of this study, future research could explore multiple dimensions to deepen our understanding of and support for the nutritional information-seeking behaviors of patients after esophageal cancer surgery. First, future studies can expand sample diversity and coverage by conducting multicenter research involving postoperative patients with esophageal cancer from different regions, hospitals of varying levels, and diverse demographic and clinical backgrounds to enhance the transferability of the findings. In addition, longitudinal qualitative studies are recommended to systematically track the dynamic trajectories of nutritional information-seeking behaviors across different postoperative stages. Such studies may help elucidate the evolution of behavioral patterns and their influencing factors, thereby providing evidence for the development of stage-specific and stratified nutritional information support programs. Future research should also include patients, family caregivers, and healthcare professionals simultaneously and integrate multiple data sources, such as patient diaries and clinical nutrition records, to obtain a more comprehensive and objective understanding of the interactions and alignment of needs among multiple stakeholders during nutritional information seeking and decision-making processes. Finally, quantitative studies are needed to validate the themes and needs identified in the present study. Integrating qualitative and quantitative findings may facilitate the development of more evidence-based and targeted nutritional information support interventions for postoperative patients with esophageal cancer.

## Conclusion

This study demonstrates that nutritional information seeking among patients recovering at home after esophageal cancer surgery is a dynamic and multifaceted process involving information acquisition, interpretation, and behavioral application. Patients commonly exhibited symptom-driven and fragmented patterns of nutritional information seeking and encountered multiple barriers, including limited access to professional guidance, difficulties in evaluating information credibility, and inconsistencies across multiple information sources. Challenges in understanding and applying nutritional information further affected the translation of information into effective self-management behaviors.

These findings suggest that nutritional support for post-esophagectomy patients should move beyond routine discharge education toward a continuous, individualized, and family-inclusive information support model. Healthcare professionals should strengthen guidance on symptom-related nutritional management, improve the accessibility and practicality of nutritional information, and support both patients and caregivers in developing the ability to identify, interpret, and apply health information effectively. Such efforts may help promote more systematic nutritional self-management and improve postoperative recovery outcomes.

## Data Availability

The original contributions presented in the study are included in the article, further inquiries can be directed to the corresponding author.
